# Single‐Nucleus Transcriptome Analysis Provides New Insights Into B Chromosome Elimination in Sorghum

**DOI:** 10.1111/pbi.70737

**Published:** 2026-08-07

**Authors:** T. Bojdová, P. Abaffy, L. Valihrach, M. Karafiátová, J. Bartoš

**Affiliations:** ^1^ Centre of Plant Structural and Functional Genomics Institute of Experimental Botany of the Czech Academy of Sciences Olomouc Czech Republic; ^2^ Department of Cell Biology and Genetics, Faculty of Science Palacky University Olomouc Czech Republic; ^3^ Laboratory of Glial Biology and Omics Technologies Institute of Biotechnology of the Czech Academy of Sciences Vestec Czech Republic

**Keywords:** B chromosome, chromosome elimination, chromosome segregation, snRNA‐seq, *Sorghum purpureosericeum*

## Abstract

B chromosomes are supernumerary entities found in many plant species, with some exhibiting tissue‐specific elimination. In *Sorghum purpureosericeum*, extensive B chromosome elimination occurs during embryogenesis affecting most of the embryonic organs, leaving the B chromosome maintained mainly in limited regions of meristems. The dynamics of the process and rarity of the transcripts associated with elimination make capturing it challenging. To address this, we performed single‐nucleus RNA sequencing (snRNA‐seq) on embryos undergoing B chromosome elimination. This approach enabled detection of more B‐linked transcripts compared to previous methods. Notably, we identified nuclei with B‐specific transcripts, which predominantly clustered in a single cluster in both replicates of B‐positive embryos. Further analysis of these clusters revealed three subpopulations with divergent transcriptional profiles. One subpopulation showed gene expression patterns suggesting active elimination of the B chromosome, while the other subpopulations are expected to exhibit regular segregation of the B chromosome and/or preparation for the elimination process. Our analysis provides resolution so far missing in current studies and highlights a clear benefit of the single‐cell approaches for studying specific behaviour of the B chromosomes.

## 1 Introduction

B chromosomes (Bs) are dispensable supernumerary elements found in about 3000 species, generally existing alongside the standard A chromosome set in all tissues (D'Ambrosio et al. 2017). However, in some species, Bs are eliminated in a tissue‐specific manner (Nygren 1957; Baenziger 1962; Mendelson and Zohary 1972; Mochizuki 1957; Karafiátová et al. 2024). Their elimination patterns are highly variable; e.g., *Poa alpina* and *Agropyron cristatum* lose Bs from adventitious roots but retain them in primary roots (Baenziger 1962) whereas *Aegilops speltoides* , *Aegilops mutica*, and *Poa timoleontis* eliminate Bs from primary roots (Nygren 1957; Mochizuki 1957; Mendelson and Zohary 1972). A particularly striking case is *Sorghum purpureosericeum*, where Bs are nearly completely lost from mature somatic tissues (Karafiátová et al. 2024), similar to germline‐restricted chromosomes (GRCs) in flies and songbirds, which are absent from all somatic cells (Hodson et al. 2021; Borodin et al. 2022).

The onset of B chromosome elimination can be traced back to embryogenesis, as seen in *Aegilops speltoides* (Ruban et al. 2020) and *Sorghum purpureosericeum* (Karafiátová et al. 2024; Bojdová et al. 2026). Bs are progressively eliminated during embryonic development and therefore these chromosomes are inherently programmed to persist only in specific tissues. Recent studies in *A. speltoides* (Boudichevskaia et al. 2020) and *S. purpureosericeum* (Bojdová et al. 2026) focused on the identification of candidate genes linked to B chromosome elimination in embryo transcriptome. However, these studies have relied on whole embryo RNA‐seq, which conceals cell‐type‐specific dynamics critical to understanding the elimination process. As embryo comprises a heterogeneous mixture of cells and only a small subset undergoes B chromosome elimination, transcripts associated with it are likely to be underrepresented in conventional transcriptomic experiments. In whole embryo RNA‐seq, the candidate transcripts tend to fade in the transcriptomes of the vast majority of cells (either with stable B chromosome or after its elimination) and it is impossible to assign them to the specific cells undergoing elimination. Single‐cell or single‐nucleus RNA sequencing (sc/snRNA‐seq) could offer a solution to this limitation by allowing the detection of rare cell populations undergoing elimination and corresponding transcripts. To date, no single‐cell transcriptomic study targeting B chromosome elimination has been conducted, largely due to the plant species pioneering the B chromosome research having Bs stably maintained across cell types. Further, this approach has neither been used for germline‐restricted chromosomes (GRCs) eliminated from somatic tissues, presenting a compelling parallel to B chromosome elimination. GRCs are much more frequent than B chromosomes undergoing elimination (Hodson et al. 2021; Borodin et al. 2022), but are challenging to study due to the difficulty of obtaining germline tissues at the appropriate developmental stages. Thus, *Sorghum purpureosericeum* offers a possibility to be a model system to investigate the molecular mechanisms underlying programmed chromosome elimination.

Importantly, the impact of B chromosome research extends beyond basic biology. B chromosomes hold massive potential in biotechnology as naturally occurring “vectors”. Being non‐essential, they can be utilised as molecular “cargo ships” to stack multiple traits without disrupting the standard genome (Houben and Schubert 2007; Liu et al. 2025). Furthermore, understanding the precise mechanisms of programmed B chromosome elimination could enable the design of advanced tissue‐specific minichromosomes (Liu et al. 2025, 2013). For instance, engineered B chromosomes could drive trait expression in vegetative tissues but undergo targeted elimination during embryogenesis or gametogenesis, providing a natural biocontainment strategy to prevent transgene escape.

In this study, we explore whether single‐nucleus RNA sequencing is beneficial to uncover cell‐to‐cell transcriptional heterogeneity associated with B chromosome elimination. By profiling embryonic tissue of *S. purpureosericeum* at 21 days after pollination (DAP), we aimed to determine whether elimination‐specific signatures can be resolved in the population of cells possessing B chromosomes. Although exploratory, our work highlights the potential of this approach to uncover cell‐specific mechanisms governing chromosome fate. The findings presented lay the groundwork for future studies and establish a conceptual framework for using sc/snRNA‐seq to investigate B chromosome‐specific phenomena at the single‐cell resolution.

## 2 Results and Discussion

### 2.1 Single‐Nucleus RNA‐Seq Offers a Promising Strategy for Solid Tissues Resilient to Protoplasting

To address the changes in the cells of wild sorghum *S. purpureosericeum* embryos, which direct the B chromosome to its elimination, we investigated the RNA‐landscape at the resolution of single nuclei. We analysed the nuclei of the embryos of approx. 21 DAP (Figure 1A,B), where the extent of the B chromosome elimination accompanying the embryo differentiation was documented (Figure 1C, Karafiátová et al. 2024; Bojdová et al. 2026). At the targeted stage, the B chromosome elimination significantly proceeded, and many embryonic tissues are free of the B chromosome. However, even cells showing signals from B‐specific probes do not exhibit stable presence of the B chromosome and its elimination continued as evidenced by the presence of micronuclei (Figure 1C,D). Embryonic nuclei suspension is thus a complex cocktail containing the nuclei lacking the B chromosome, harbouring the B chromosome and undergoing the B chromosome elimination (Figure 1E). We have analysed two biological replicates of embryos with B chromosomes (+B) and two B chromosome‐free (0B) controls.

**FIGURE 1:**
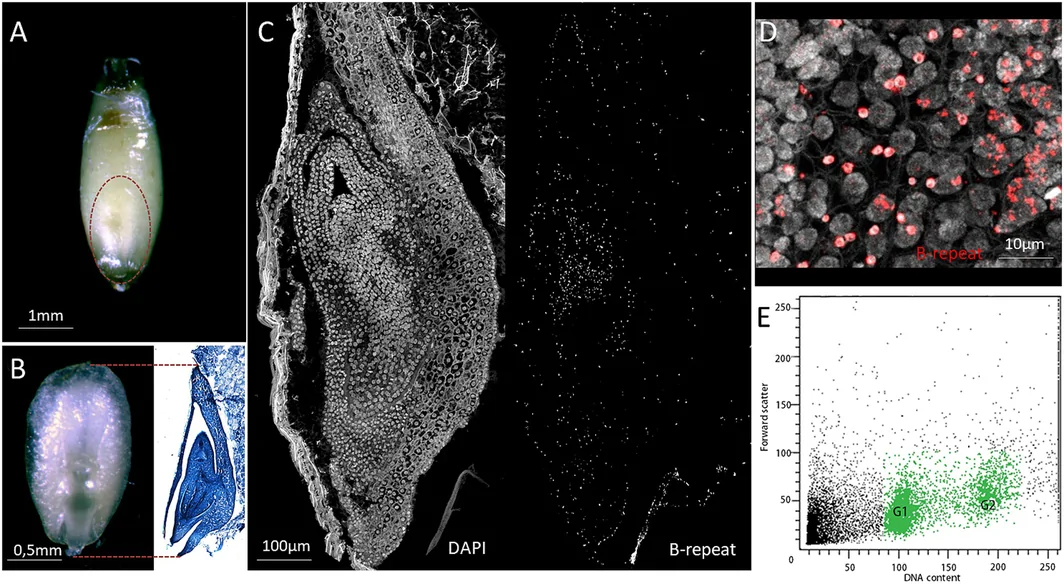
Embryo anatomy and progress of the B chromosome elimination in wild sorghum *S. purpureosericeum*. (A) Developing seed of wild sorghum approx. 21DAP. Embryo position is indicated by red‐dashed line. (B) Intact isolated embryo of 21 DAP (ventral side) and its transversal section stained with toluidine blue illuminating embryo organogenesis. (C) Distribution of the B chromosome in developing embryo visualised using FISH with B‐specific probe cocktail on embryo cryo‐sections; DAPI counterstaining (left) and signal from B‐repeat cocktail (right). (D) Chimeric composition of embryonic tissue consisting of three types of nuclei—harbouring B chromosomes (positive for B‐repeat signal), undergoing B chromosome elimination (B chromosome located in micronucleus) and yet free from B chromosome (nuclei absent for specific signal). B chromosome is detected using FISH with B‐repeat cocktail (red). (E) Nuclei isolation for snRNA‐seq using flow cytometry. Green‐marked populations represent the mix of +B and 0B embryonic nuclei in G1/S/G2 phase used as input DNA for sequencing library.

A total of 30 000 nuclei were collected through flow sorting per each replicate (Figure 1E). The nuclei formed two distinct populations in flow cytometric analysis differing in ploidy level corresponding to cell cycle phases (G1 and G2) and not by the B chromosome presence/absence (Figure 1E). Both populations as well as nuclei with intermediate DNA content (S phase) were profiled via droplet‐based snRNA‐seq. In pre‐processing steps, 6.7% reads were removed as ambient RNA, and 4.2% cells were removed as potential doublets on average per replicate. After this data filtering, 4445 to 9445 nuclei were retained for further analysis. On average, 2005–2431 genes per nucleus were detected, with a mean read depth of 19 655 reads mapped to genes and an average unique molecular identifier (UMI) count of 3067–4266 per cell (Source data: Table S1). In total, expression of 34 700–37 066 genes was recorded within all cells per replicate, representing an improvement over the 30 580 genes reported in the previous RNA‐seq study (Bojdová et al. 2026). Importantly, detection of expressed B chromosome genes also increased, from 2012 to 2975.

Candidate genes controlling the B chromosome elimination in *S. purpureosericeum* have been identified previously (Bojdová et al. 2026). However, the analysis was conducted on whole embryos, disabling the spatial tracking candidate gene expression. In contrast, single‐cell RNA sequencing (scRNA‐seq) would enable such resolution, nevertheless protoplasting embryo samples for scRNA‐seq presents technical challenges. To circumvent these difficulties, we opted for single‐nucleus RNA sequencing (snRNA‐seq), which sequences nuclear transcripts, thereby eliminating the need for protoplasting. This approach has been successfully applied in plant embryo transcriptomics studies (Kao et al. 2021; Long et al. 2021). In both studies, the reported gene detection metrics were comparable with those obtained in our experiment. Considering the pros and cons of the method, the RNA‐analysis at the single nuclei resolution represents a meaningful compromise yet providing valuable knowledge. Our experiment shows a clear benefit of this method and outlines a new trend in the study of B chromosome elimination.

### 2.2 Functional Assignment of snRNA‐Seq Clusters Identified Population of Nuclei Harbouring the B Chromosome

Unsupervised clustering analysis using Seurat software (Hao et al. 2024) identified distinct embryonic cell populations based on their nuclear transcriptomic profiles. Visualisation of the data via uniform manifold approximation and projection (UMAP) (Becht et al. 2019) of the +B_1, +B_2, 0B_1 and 0B_2 replicates revealed 16, 12, 12 and 13 cell populations at the 0.5 resolution, respectively (Figures 2A and S1–S4), with cluster sizes ranging from 43 nuclei (cluster 15 in +B_2) to 1469 nuclei (cluster 0 in 0B_2). A total of 4163, 3785, 3492 and 3816 genes were assigned as marker genes (using default settings) for +B_1, +B_2, 0B_1 and 0B_2 replicates, respectively (Figure 2B, Source Data: Table S2–S5). A classification of the clusters was performed using data from the +B_1 replicate, the analysis of which yielded the largest number of clusters. To annotate clusters, we firstly took advantage of published embryonic single‐cell transcriptomes of barley (Peirats‐Llobet et al. 2023) and maize (Wu et al. 2025) comparing *S. purpureosericeum* marker genes to their homologues in these datasets. Additionally, we searched for marker genes in scPlantDB (He et al. 2024), a database of scRNA‐seq studies, and examined the previously characterized biological functions of the identified cluster‐specific marker genes. This strategy enabled the classification of four major nuclei groups comprising 10 out of 16 clusters for the +B_1 replicate: embryo (emb‐1 to emb‐6), scutellum (scu), endosperm (end) and actively proliferating cells (S, G2/M).

**FIGURE 2:**
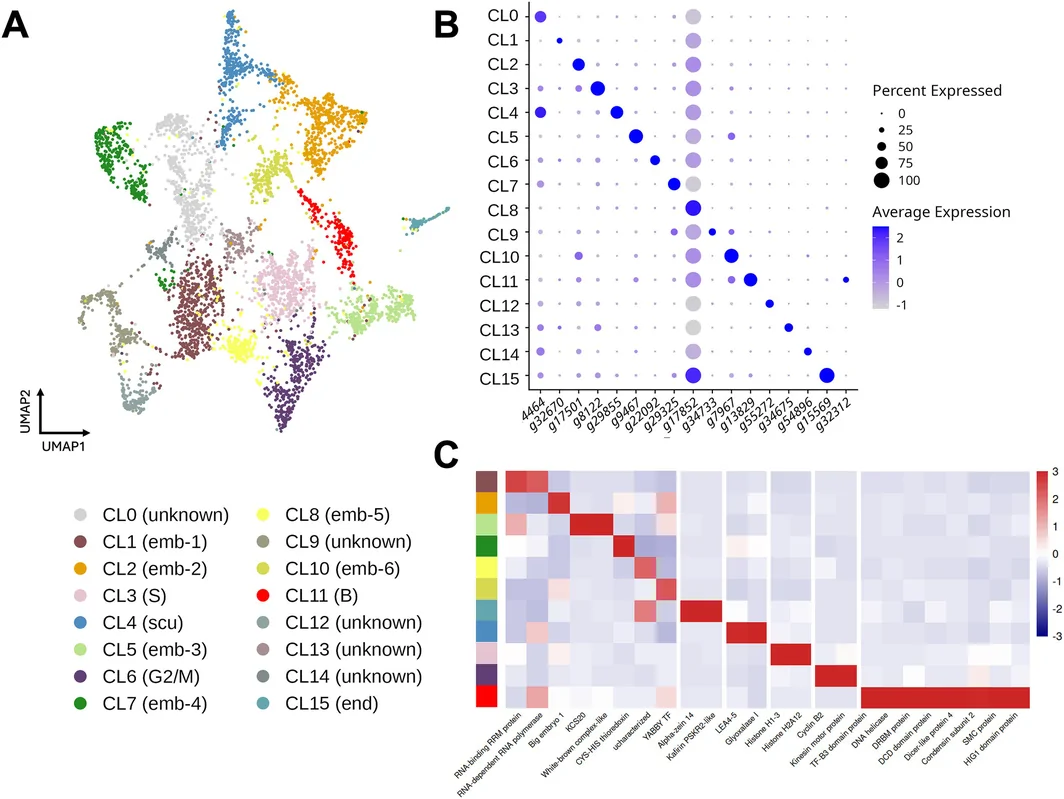
Single‐nucleus RNA‐seq clustering and cluster annotation of *Sorghum purpureosericeum* embryo tissues. (A) UMAP visualisation of 16 snRNA‐seq clusters from 5029 nuclei of +B_1 replicate. Each dot indicates a single nucleus. (B) Expression dotplot of cluster‐specific top marker genes for each cell type. The last gene (g32312) represent a marker gene used for validation of the B cluster identity using in situ localization. Dots size highlights proportion of cells expressing given marker genes in each cluster and dots colour corresponds to mean expression in all cells in given cluster. (C) Heat map of average SCT‐transformed expression of annotated marker genes across clusters. First column represents colour codes for the clusters identical to panel A.

Among clusters assigned to the embryo, CL1 (emb‐1) expressed root specific β‐1,3‐glucanases (Doxey et al. 2007). CL2 (emb‐2) included a marker identified as a trans‐Golgi localised transporter required for regulation of lateral organ size and initiation rate in plants (Suzuki et al. 2015). CL5 (emb‐3) was characterized by epidermal markers, including 3‐ketoacyl‐CoA synthase 20 (KCS20), which is involved in cuticular wax biosynthesis (Suh et al. 2005; Wang et al. 2023), as well as a *white‐brown complex‐like protein* linked to cutin metabolism (*ABCG*) (Panikashvili et al. 2010). CL7 (emb‐4) was identified as a vascular cells cluster, as it expressed marker genes atypical Cys/His‐rich thioredoxin and embryonic cell protein 63 (Schmid et al. 2005). CL8 (emb‐5) was assigned to the embryo based on its association with embryonic clusters. CL10 (emb‐6) was associated with leaf development, marked by a *YABBY family transcription factor*, which is also a known maize leaf marker (Wu et al. 2025).

CL15 (end) represented residual endosperm. Marker genes supporting that included homologues of *kafirin PSKR2‐like*, an endosperm storage protein (Li et al. 2024) and *alpha‐zein 14*, a well‐established maize endosperm marker (Wu et al. 2025). In the scutellum‐associated cluster CL4 (scu), key markers included *Late Embryogenesis Abundant protein (LEA‐4‐5), metallothionein‐like protein*, and *glyoxalase I family protein (GLO1)*, all of which have been identified in the barley scutellum (Peirats‐Llobet et al. 2023). Two clusters (CL3 and CL16) were identified as proliferating meristematic cell populations, one corresponding to cells in the S phase and the other in the G2/M phase of the cell cycle. The CL3 (S phase) was characterized by the expression of *histones H1.3* and *H2AC12*, as well as *SMC3*, consistent with previously identified S‐phase markers (Zhang et al. 2021). CL6 (G2/M) was marked by *cyclin B2 (CCNB2), aurora1, and kinesin motor family* proteins, which have been identified as G2/M markers in the Arabidopsis shoot apex (Zhang et al. 2021). Clusters CL0, CL9, CL12, CL13, and CL14 were missing marker genes with clear functional/morphological characteristics, and we were therefore unable to confidently assign them to any specific tissue or functional group at this stage. Because our annotation strategy relied on homologous marker genes from other species, these unassigned clusters may represent *S. purpureosericeum*‐specific embryonic states where cross‐species ortholog mapping lacks sufficient resolution. The clusters of remaining replicas were annotated based on the +B_1 marker set and MetaNeighbor clustering results (Figures S2–S4, Figure S5A–C).

In addition to described annotated groups, cluster 11 in +B_1 replicate (203 nuclei) did not fall into any of the above categories based on its transcriptional profile. Due to its distinct expression signature enriched for B chromosome‐encoded genes, we refer to it as the cluster of nuclei comprising B chromosome. Similarly, cluster 9 in +B_2 replicate (163 nuclei) exhibited a similar expression pattern (Figure S2). At this late developmental stage—approximately 3 weeks after fertilisation—the B chromosome is retained in the limited number of cells. Given that only a fraction of nuclei in the embryo are derived from B chromosome‐containing proliferative tissue, the relative size of the B chromosome cluster (∼4% of all nuclei) is consistent with the expected proportion of those cells within the embryo.

### 2.3 snRNA Approach Resolves the Complexity of Embryonic Tissue With Respect to the B Chromosomes

In total, 37 066 genes were detected as expressed in the snRNA‐seq dataset, including 2975 genes located on the B chromosome. B chromosome‐encoded genes were detected across all identified clusters; however, the number of B genes expressed per nucleus varied among clusters. Cells/nuclei in cluster 11 and cluster 9 in +B_1 and + B_2 replicate, respectively, expressed a higher number of genes encoded by the B chromosome (Figure S6). This suggests that, while nuclei harbouring B chromosomes were distributed across all clusters, nearly all nuclei in mentioned clusters carried the B chromosomes in +B samples.

We experimentally validated cluster CL11 in +B_1 replicate as cluster containing B‐positive nuclei using HCR Gold RNA‐FISH. The RNA probe was designed to the transcript of the B‐encoded gene g32312 representing marker gene in CL11, which was predicted to comprise B‐carrying nuclei based on *in silico* analysis of captured transcripts. Hybridization of g32312 HCR HiFi probe on longitudinal embryo cryosection confirmed that transcript identified in cluster 11 is present in cells harbouring B chromosome in sorghum embryo (Figures 3C and S7). The signal was observed in a limited group of cells in the region of the plumule (Figure 3C), while missing in remaining embryonic tissues (Figure S7). This transcript localization mirrors DNA FISH results with B‐specific repeat defining the area of the embryo where cells maintain the B chromosome (Figure 1C). The specificity of the transcript of g32312 to the B chromosome‐harbouring cells was further supported in an independent experiment on B chromosome‐absent embryo section, which clearly resulted in no hybridization signal from g32312 RNA probe (Figure S8).

**FIGURE 3:**
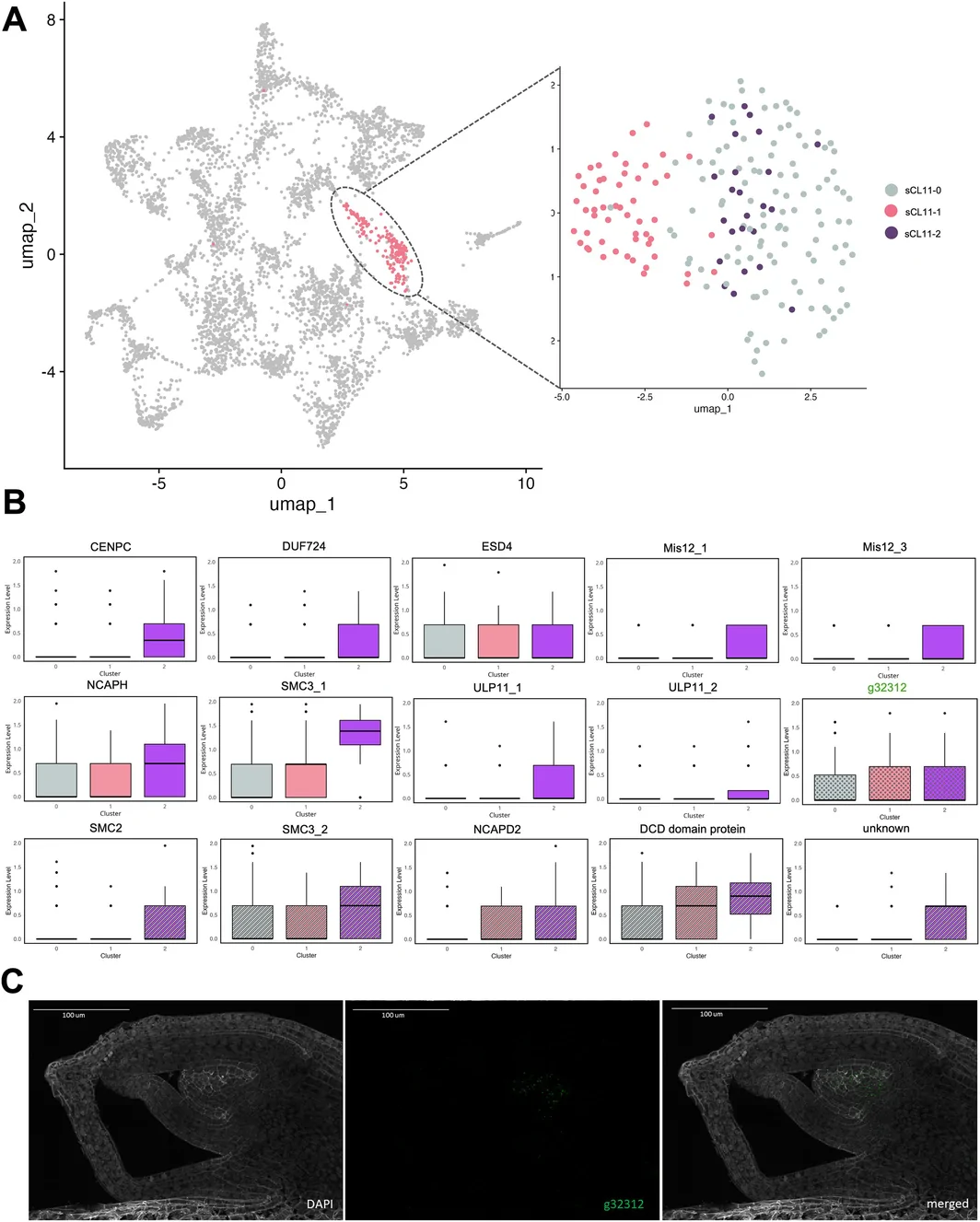
Analysis of a cluster associated with B chromosome elimination candidate gene expression. (A) Left: UMAP representation of all captured single nuclei in +B_1 sample, as shown in Figure 2, highlighting the B cluster (pink). Right: UMAP displaying independent clustering of the nuclei in the B cluster, with resolution set to 0.8. (B) Boxplots depicting the expression levels of B chromosome elimination candidate genes in the B cluster (note elevated expression of candidate genes in subcluster sCL11‐2). Boxplots for candidate genes with no detectable expression were omitted. Another boxplot shows the expression of the g32312 marker gene (highlighted in green), selected for validation of the B chromosome cluster (note expression of the gene across all three subclusters). Boxplots with stripe pattern highlight newly identified B chromosome elimination candidate genes. (C) Experimental validation of the (cluster CL11) using HCR RNA‐FISH targeting the transcript of B‐encoded marker gene g32312 (green) in longitudinal embryo sections. Signal is restricted to a limited group of cells in the plumule region, while absent from surrounding embryonic tissues.

Cluster 11 was further characterized by the presence of 94 marker genes, of which 55 were located exclusively on the B chromosome (Source data: Table S6). Of these 55 genes, 3 had been previously identified in an RNA‐seq study as significantly upregulated in B chromosome possessing embryo (Bojdová et al. 2026). Cluster 9 of +B_2 sample comprised a total of 186 marker genes, 55 of which were located on the B chromosome (Source data: Table S7); 4 of these had been previously identified in the same study. This indicates that cluster 11 is distinctly enriched for B chromosome transcriptional signatures. Interestingly, it was not possible to align cluster 11 to any of the embryo tissue, likely due to the B‐cluster complexity.

Single‐nucleus RNA‐seq outperformed whole embryo RNA‐seq by detecting a larger number of expressed genes, including 2975 located on the B chromosome, many of which were missed or underrepresented in conventional RNA‐seq. Moreover, the transcriptional dominance of B chromosome marker genes caused B‐positive nuclei to cluster together. This clustering makes nuclei with B chromosomes easier to identify, enabling the targeted investigation of both known and novel B chromosome transcripts.

### 2.4 Subclustering of B Chromosome Possessing Cells Indicates Those Undergoing Elimination of B Chromosome as Well as Novel Candidate Genes

While investigating the expression of previously identified 28 candidate genes associated with B chromosome elimination (Bojdová et al. 2026), we identified the B chromosome variant of *Structural Maintenance of Chromosomes 3* (*SMC3*) and Non‐SMC *Condensin I Complex Subunit H* (*NCAPH*) as strong marker genes for the B cluster (CL11 in +B_1) (Source Data: Table S6). SMC3, together with SMC1, forms the structural backbone of the cohesin ring complex, which encircles sister chromatids and prevents premature separation before anaphase. This cohesion is established during DNA replication in the S phase and persists until anaphase, when separase cleaves the cohesion complex to release the chromatids. NCAPH is a regulatory subunit of the condensin I complex, which is essential for chromosome condensation during mitosis and meiosis. Unlike the cohesin complex, which maintains chromatid cohesion, the condensin complex is responsible for compacting chromosomes to ensure their proper segregation. To better resolve transcriptional differences within this cluster, we performed independent clustering on nuclei contributing to CL11 in +B_1 sample. This approach allowed us to focus on subtle variations within the B cluster (Figure 3A,B).

For the CL11‐specific analysis, we applied the same Seurat processing pipeline as for the full dataset but adjusted the clustering resolution to 0.8, which resulted in the three subclusters. The most interesting sCL11‐2 was defined by 126 marker genes (Source data: Table S6), including 55 genes localised to the B chromosome, among them *SMC3* and *NCAPH*. Additionally, sCL11‐2 exhibited expression of other candidate genes encoded by the B chromosome—*CENP‐C* and two copies of *Mis12* (*Mis12_1* and *Mis12_2*), which are involved in kinetochore function, as well as *SUMO‐specific protease ESD4* and a *DUF724 domain‐containing protein* (Figure 3B). In contrast, subcluster sCL11‐0 was defined by 22 marker genes, out of which only 3 were located on the B chromosome, and subcluster sCL11‐1 by 245 marker genes with only 9 B located genes. Despite the lack of B chromosome‐encoded genes among its top differentially expressed marker genes, sCL11‐0 and sCL11‐1 still showed clear expression of B chromosome genes. Considering the expression of B genes, all three subclusters likely contain nuclei harbouring B chromosomes. However, the transcriptional profile of sCL11‐0 lacks expression of candidate elimination‐associated genes such as *Mis12* paralogs or *SMC3*, indicating that cells in this cluster may represent a population with stable B chromosomes. In contrast, sCL11‐2 may correspond to a subset of B chromosome‐containing cells actively undergoing, or primed for, the elimination process. The subcluster sCL11‐1 either represented nuclei with intermediate expression of candidate genes (expressing only some of them) or collected nuclei preparing for elimination, but with lower RNA quality compared to subcluster sCL11‐2. Several additional marker genes of sCL11‐2 followed the expression patterns of these B chromosome elimination candidates (Figure 3B). Previously identified components of the condensin complex—subunits 2 and 3 (Bojdová et al. 2026)—were enriched of subunit 1 (*NCAPD2*; this study). More novel candidates, including *histone deacetylase 15‐like* (*HDA15‐like*), DCD domain containing protein, an additional copy of *SMC3 and SMC2*, showed the same expression pattern as well as numerous yet uncharacterized transcripts (Figure 3B). Similar patterns of expression for candidate genes were observed also after subclustering of the CL9 in +B_2 sample (Figure S9).

*SMC3*, *Mis12*, and condensin subunits have previously been associated with transcriptomic analyses of embryos undergoing B chromosome elimination in both *S. purpureosericeum* (Bojdová et al. 2026) and *Aegilops speltoides* (Boudichevskaia et al. 2020). Moreover, cohesion was previously discussed in the context of nondisjunction in rye (Banaei‐Moghaddam et al. 2012; Chen et al. 2024), maize (Han et al. 2007), and *Aegilops speltoides* (Wu et al. 2019). Thus, identification of the same genes in our snRNA study suggests that these genes may contribute to the elimination process.

Interestingly, sub‐clustering within cluster CL11 and CL9 (in +B_1 and + B_2 replicate, respectively) clearly highlighted the advantage of the single‐nucleus approach. In whole embryo RNA‐seq analysis, which strictly distinguishes samples as +B and 0B, all differentially expressed genes are attributed to the presence of the B chromosome. Contrary, the different transcriptional profiles of +B nuclei can be traced using snRNA‐seq, indicating their different fates. While the nuclei in subcluster sCL11‐0 may further maintain the B chromosome, the genes transcribed in cells contributing to sCL11‐2 likely suggest the onset of the elimination. This approach enabled us to track known elimination candidates and to identify previously unrecognised ones within the eliminating subcluster sCL11‐2.

Based on snRNA‐seq data analysis in *Sorghum purpureosericeum* embryos, this study for the first time provided insights into the process of B chromosome elimination at the single‐nucleus resolution. This approach captured substantially more B chromosome transcripts than conventional RNA‐seq and revealed that their expression is so dominant that nuclei harbouring B chromosomes cluster together. Further, it uncovered the identity of the cells in which the B chromosome is eliminated and identified additional candidate genes potentially involved in the process. The high resolution of our analysis provides a unique perspective on the dynamics of the elimination process and demonstrates that the transcriptomes of cells with different B chromosome fates can differ significantly. Ultimately, understanding the molecular mechanisms driving B chromosome behaviour and tissue‐specific elimination not only sheds light on genome evolution but also provides a blueprint for engineering the next generation of smart, tissue‐restricted plant minichromosomes.

## 3 Methods

### 3.1 Plant Material and Cultivation

The progeny of *Sorghum purpureosericeum* plants possessing (+B) and lacking (0B) B chromosomes were harvested at the Institute of Experimental Botany (Czech Republic) and used as plant material. Plants were grown under short‐day conditions, featuring 10 h of daylight, with daytime temperatures maintained at 29°C and nighttime temperatures at 25°C. The presence of the B chromosome in plants was scored in the initial spike using flow cytometry, following the methodology published previously (Karafiátová et al. 2021).

### 3.2 Embryo Harvesting, Sectioning and in Situ Hybridization

All embryos were collected from +B plants and, in parallel, from 0B plants lacking B chromosomes. The stage of embryo development was determined as described in Bojdová et al. (2026). Embryos at stage of ~21 DAP were further screened for B chromosome presence, which was assessed in endosperm using B chromosome‐specific PCR marker SpuB (Karafiátová et al. 2024). Both +B and 0B embryos were selected for sectioning and snRNA‐seq.

Simultaneously, two protocols for embryo‐sectioning were employed. First, wax‐sectioning coupled with toluidine blue staining was used to visualise the anatomy of the embryos. Second, the localization of the B chromosome in embryonic tissues was detected by fluorescent in situ hybridization on embryo cryo‐sections. Both protocols for sectioning together with probe labelling and FISH followed the methodologies described in Karafiátová et al. (2024) and Bojdová et al. (2026).

### 3.3 Preparation of Nuclei Suspension and Flow Cytometric Purification

About 20 deep frozen +B or 0B embryos per replicate were pooled and used for nuclei isolation. The individual replicates are labelled +B_1 and + B_2 for embryos possessing the B chromosome; and 0B_1 and 0B_2 for embryos lacking the B chromosome throughout the manuscript. Sorghum nuclei suspension was prepared in 0.5 mL of Galbraith buffer with RNase inhibitor Ultima H (0.5 U/μL)(Diana Bitechnologies) using glass tissue grinder (KimbleKontes) with glass pestle size A. Embryos were homogenised for 4 min and crude nuclei suspension was filtered through 20 μm nylon mesh. Residual nuclei in tissue grinder were washed out with another 0.5 mL of Galbraith buffer with RNase inhibitor and filtered. Embryonic nuclei were DAPI stained (2 μg/mL) and analysed using cell sorter FACS Aria Fusion (BD Biosciences, San Jose, CA, USA). In total, 30 000 of G1, S and G2 population of embryonic nuclei per each sample (Figure 1E) were purified into 1 mL of 1× PBS with 1% BSA and RNase inhibitor (0.5 U/μL).

### 3.4 Single‐Nuclei RNA‐Seq Library Preparation and Sequencing

Sequencing library was prepared using the GEM‐X Universal 3′ Gene Expression v4 4‐plex On‐Chip Multiplexing (OCM) Kit (10× Genomics, Pleasanton, CA, USA; Cat. No. 1000779) according to the manufacturer's instructions. Briefly, four nuclei suspensions were loaded individually into the four inlet wells of the GEM‐X 3′ OCM Chip Kit, loading approximately 10 000 nuclei per sample. Partitioning and on‐chip multiplexing were performed on the Chromium X instrument (10× Genomics); samples were co‐partitioned in a single GEM recovery well and subsequently demultiplexed computationally during data analysis based on sample‐specific OCM barcodes. Before sequencing library construction, cDNA was amplified in 11 PCR cycles, yielding ~29.3 ng/μL of amplified cDNA. After fragmentation and adaptor ligation, final libraries were indexed using the Dual Index Kit TT, Set A (10× Genomics) in 11 PCR cycles. The quality of the final library was controlled by Fragment Analyser (Agilent) using the High‐Sensitivity NGS Fragment Kit. The library was sequenced on the Element Biosciences AVITI24 system using one lane of a Cloudbreak Freestyle High output flow cell (2 × 75 bp) with the read configuration: 10‐10‐28‐134 cycles.

### 3.5 snRNA‐Seq Data Mapping and Pre‐Processing

snRNA‐seq data were analysed using the *S. purpureosericeum* reference sequence and annotation published recently (Bojdová et al. 2026). The annotation was further modified with peaks2UTR pipeline, which corrected missing 3′UTR to increase the number of detected genes (Haese‐Hill et al. 2023). Reads were mapped to the reference sequence separately using STAR 2.7.11b with sample‐specific OCM barcodes IDs (OB1–OB4). The maximum intron length was set to 50 000 bases and multimapping limited to 15 loci. Cell barcodes and UMIs were processed using soloType CB_UMI_Simple with a 16 bp cell barcode (‐‐soloCBstart 1 ‐‐soloCBlen 16) and a 12 bp UMI (‐‐soloUMIstart 17 ‐‐soloUMIlen 12). UMI deduplication was performed using 1MM_Directional, and multi‐gene UMIs were filtered (MultiGeneUMI). Reads were output as a sorted BAM file, and count matrices (features, barcodes, matrix) were generated using ‐‐soloFeatures GeneFull. Rather than computationally forcing the integration of all four biological replicates into a single manifold—which our MetaNeighbor analysis demonstrated dilutes the rare B‐specific transcriptional signatures (Figure S5A)—we analysed the replicates individually to maximise the preservation of the B‐chromosome clusters. We applied emptyDrops from the DropletUtils package (version 1.6.1) to distinguish true cells from empty droplets (Griffiths et al. 2018; Lun et al. 2019) with a lower threshold of 500 total counts and an FDR cutoff of 0.001, while doublets were detected and removed using scDblFinder (v1.16.0) (Germain et al. 2021). Ambient RNA contamination was corrected using SoupX (v1.6.2) by estimating contamination levels with autoEstCont (Young and Behjati 2020).

### 3.6 Clustering and Cell‐Type Annotation

For clustering, the Seurat package (v.5.5.0) was used (Hao et al. 2024, 2021; Stuart et al. 2019; Satija et al. 2015). Data were normalised using the SCTransform function (parameters: return.only.var.genes = TRUE, method = “glmGamPoi“, variable.features.*n* = NULL) (Hafemeister and Satija 2019). Dimensionality reduction was performed with RunPCA on all genes, and the top 30 principal components were used for Uniform Manifold Approximation and Projection (UMAP) and clustering of nuclei. Generation of spot clusters was made using the ‘FindNeighbors’ (settings: reduction = ‘pca’, dims = 1:30) followed by the ‘FindClusters’ functions in Seurat. Cluster resolution was set to 0.5. To identify marker genes for each cluster, pair‐wise comparisons of individual clusters against all other clusters were performed using the ‘FindAllMarkers’ function (settings: min.pct = 0.25, only.pos = T). To characterize marker genes, we searched for orthologous genes in maize, barley, and sorghum at https://plants.ensembl.org/ and annotated embryo cell types using previously curated marker transcripts resulting from scRNA‐seq analyses (He et al. 2024). Moreover, MetaNeighbor (v 1.26.0) was further used to quantify transcriptional similarity between clusters, enabling robust assessment of cell type correspondence across replicates. To inspect clusters B in detail, we subsetted the B cells from the SCT normalised Seurat object and performed the pipeline from RunPCA as previously described. The resolution was set to 0.8 based upon closer examination of cluster trees.

### 3.7 Experimental Validation of B‐Cluster Marker Gene Using HCR Gold RNA‐FISH

We localised the transcript from the cluster containing B‐harbouring nuclei on thin B‐positive embryo cryosections using HCR Gold RNA‐FISH kit (Molecular Instruments, USA). In paralel, 0B embryos were used as a negative control. The +B and 0B embryos were treated and sectioning was done as described in Karafiátová et al. (2024). The in situ localization was performed on freshly prepared sections stored at −80°C overnight. The marker gene g32312 of CL11 in +B_1 replicate (encoded by the B chromosome) was selected to prove the B‐profile of the nuclei in the cluster. We used the transcript of the gene to design the probe for HCR (hybridization chain reaction) RNA‐FISH. The HCR HiFi probe design as well as probe synthesis was provided by Molecular Instruments (Los Angeles, CA, USA). HCR HiFi probe X1 was designed for 4 binding sites and signal was amplified using amplifier X1 with 488 label. Sample pre‐processing, probe hybridization and signal amplification was done according to manufacturer's protocol available online (https://files.molecularinstruments.com/HCR%E2%84%A2‐Gold‐RNAFISH‐User‐Guide.pdf). Signals were observed using the Leica TCS SP8 STED 3X confocal microscope (Leica Microsystems, Wetzlar, Germany), equipped with an HC PL APO CS2 100×/1.44 Oil objective, Hybrid detectors (HyD), and the Leica Application Suite X (LAS‐X) software version 3.5.5 with the Leica Lightning module (Leica, Buffalo Grove, IL, USA).

## Funding

The work was supported by the Czech Science Foundation (grant no. 22‐02108S), the Institute of Biotechnology of the Czech Academy of Sciences (RVO: 86652036) and the Ministry of Education Youth and Sports (grants no. LUC24010 and CZ.02.01.01/00/23_020/0008540).

## Conflicts of Interest

The authors declare no conflicts of interest.

## Supporting information


**Table S1:** Quality control statistics of sorted nuclei across all snRNA‐seq samples.
**Table S2:** Differentially expressed marker genes for each cluster in the +B_1 replicate.
**Table S3:** Differentially expressed marker genes for each cluster in the +B_2 replicate.
**Table S4:** Differentially expressed marker genes for each cluster in the 0B_1 replicate.
**Table S5:** Differentially expressed marker genes for each cluster in the 0B_2 replicate.
**Table S6:** Differentially expressed marker genes for +B_1‐CL11 subclusters.
**Table S7:** Differentially expressed marker genes for +B_2‐CL9 subclusters.

**Figure S1:** Uniform Manifold Approximation and Projection (UMAP) plot of single‐nucleus data from the biological replicate +B_1.16 clusters were generated using a resolution of 0.5. Cells are coloured according to cluster annotations.
**Figure S2:** UMAP plot of single‐nucleus data from the biological replicate +B_2.12 clusters were generated using a resolution of 0.5. Cells are coloured according to cluster annotations.
**Figure S3:** UMAP plot of single‐nucleus data from the biological replicate 0B_1.12 clusters were generated using a resolution of 0.5. Cells are coloured according to cluster annotations.
**Figure S4:** UMAP plot of single‐nucleus data from the biological replicate 0B_2.13 clusters were generated using a resolution of 0.5. Cells are coloured according to cluster annotations.
**Figure S5:** MetaNeighbor analysis of replicates using shared highly variable genes. Cell type similarity across four biological replicates was assessed using MetaNeighborUS with a shared set of global highly variable genes (HVGs). The resulting AUROC similarity matrix, representing the degree of cell type correspondence across replicates, was visualised as a heatmap. The heatmap colour scale represents AUROC values, with red indicating higher similarity (higher AUROC scores) and blue indicating lower similarity (lower AUROC scores). (A) Full analysis including all four replicates. (B) Two control 0B replicates. (C) Two +B replicates. Notably, the B‐associated clusters CL11 in +B_1 and CL9 in +B_2 show strong mutual similarity, where B+ samples are analysed separately, but this relationship is not retained in the combined four‐replicate analysis (A) indicating that this clustering signal is specific to the B+ context and is diluted when all replicates are analysed together.
**Figure S6:** Distribution of B gene expression per cell across Seurat clusters in replica +B_1 (A) and +B_2 (B). Each dot represents a single cell, coloured by cluster identity and ordered by cluster along the y‐axis. The x‐axis shows the number of high confidence B genes with detectable expression (non‐zero SCT values) per cell.
**Figure S7:** Whole +B embryo cryosection with gene transcript g32312 hybridised. (A) embryo counterstained with DAPI, (B) detection of 488‐labelled RNA probe, (C) merged picture of both channels.
**Figure S8:** Localization of B‐gene transcript g32312 on cryosections of embryos lacking B chromosome (negative control). Upper panel: whole embryo section. Lower panel: detail of aerial embryonic organs (area of interest in +B embryos) indicated by yellow dash line on picture A. (A) and (D) embryo counterstained with DAPI, (B) and (E) detection of 488 labelled RNA probe, (C) and (F) merged picture of both channels.
**Figure S9:** Analysis of a cluster associated with B chromosome elimination candidate gene expression. (A) Left: UMAP representation of all captured single nuclei in +B_2 sample, as shown in Figure S2, highlighting the B cluster (pink). Right: UMAP displaying independent clustering of the nuclei in the B cluster, with resolution set to 0.8. (B) Boxplots depicting the expression levels of B chromosome elimination candidate genes in the B cluster (note elevated expression of candidate genes in subcluster sCL11‐2). Boxplots for candidate genes with no detectable expression were omitted Another boxplot shows the expression of the g32312 marker gene (highlighted in green), selected for validation of the B chromosome cluster (note expression of the gene across all three subclusters). Boxplots with stripe pattern highlight newly identified B chromosome elimination candidate genes across subclusters.

## Data Availability

Raw snRNA‐seq reads are available from the European Nucleotide Archive (ENA) under project PRJEB97401 (run no. ERR17525476). Genome assembly originally published in Bojdová et al. (2026) is available in ENA under ID—GCA_964647645.
