# Supplementary material for: Single‐Nucleus Transcriptome Analysis Provides New Insights Into B Chromosome Elimination in Sorghum
Source: Plant Biotechnol J. 2026 Aug 7;24(11):5719–28. doi: 10.1111/pbi.70737 (PMC13448169; doi:10.1111/pbi.70737)
Supplement: Supplementary file 1 [file PBI-24-5719-s001.pdf]

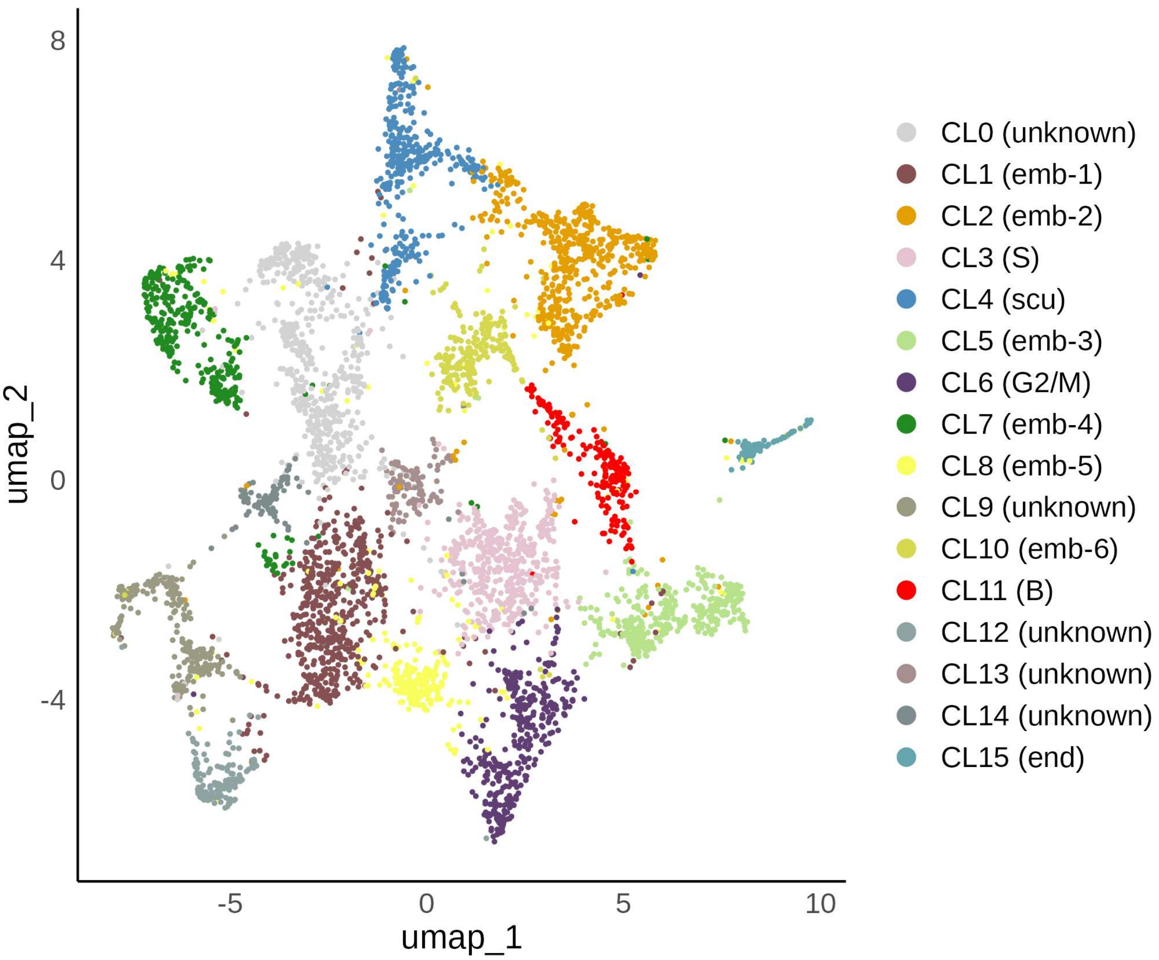

Supplementary figure S1: Uniform Manifold Approximation and Projection (UMAP) plot of single-nucleus data from the biological replicate +B_1. 16 clusters were generated using a resolution of 0.5. Cells are colored according to cluster annotations.

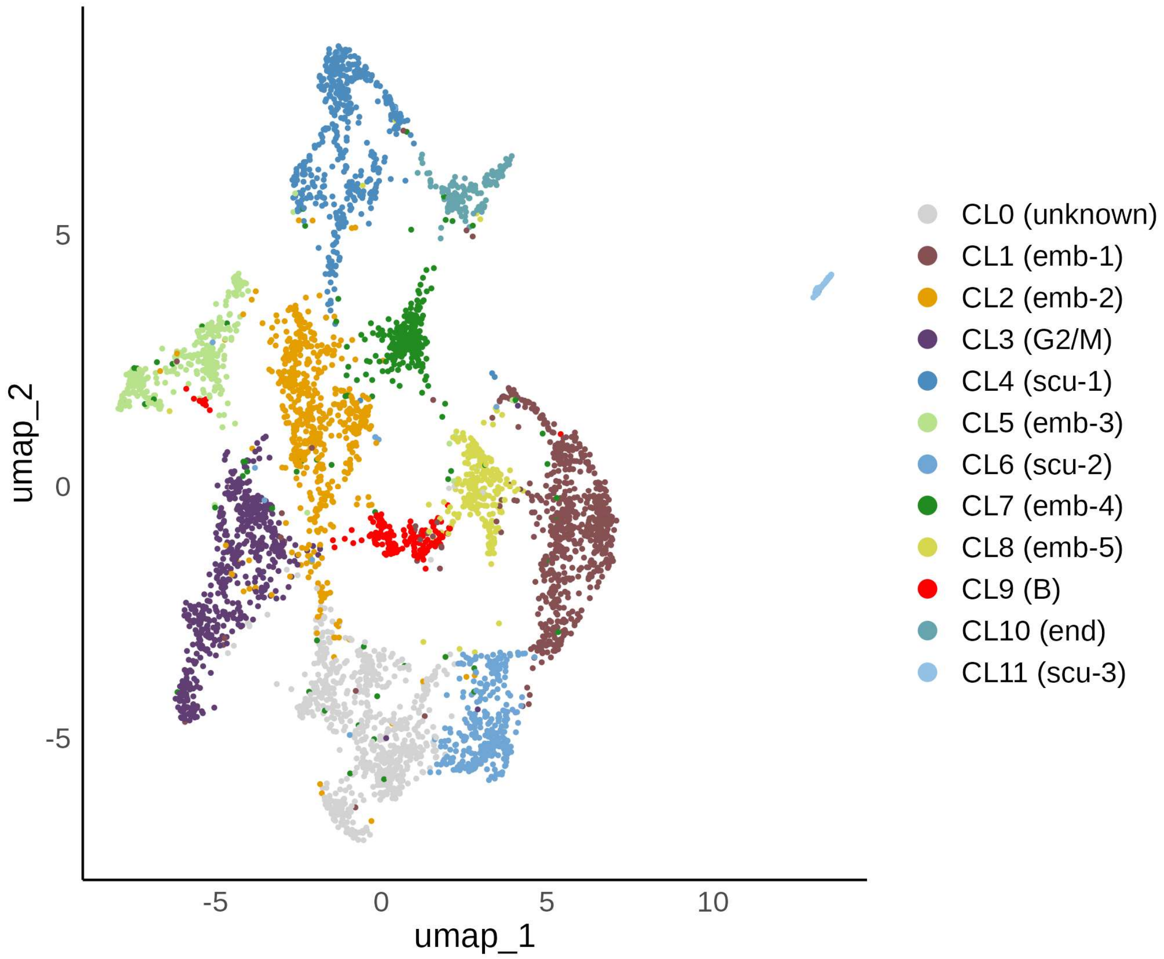

Supplementary figure S2: UMAP plot of single-nucleus data from the biological replicate +B_2. 12 clusters were generated using a resolution of 0.5. Cells are colored according to cluster annotations.

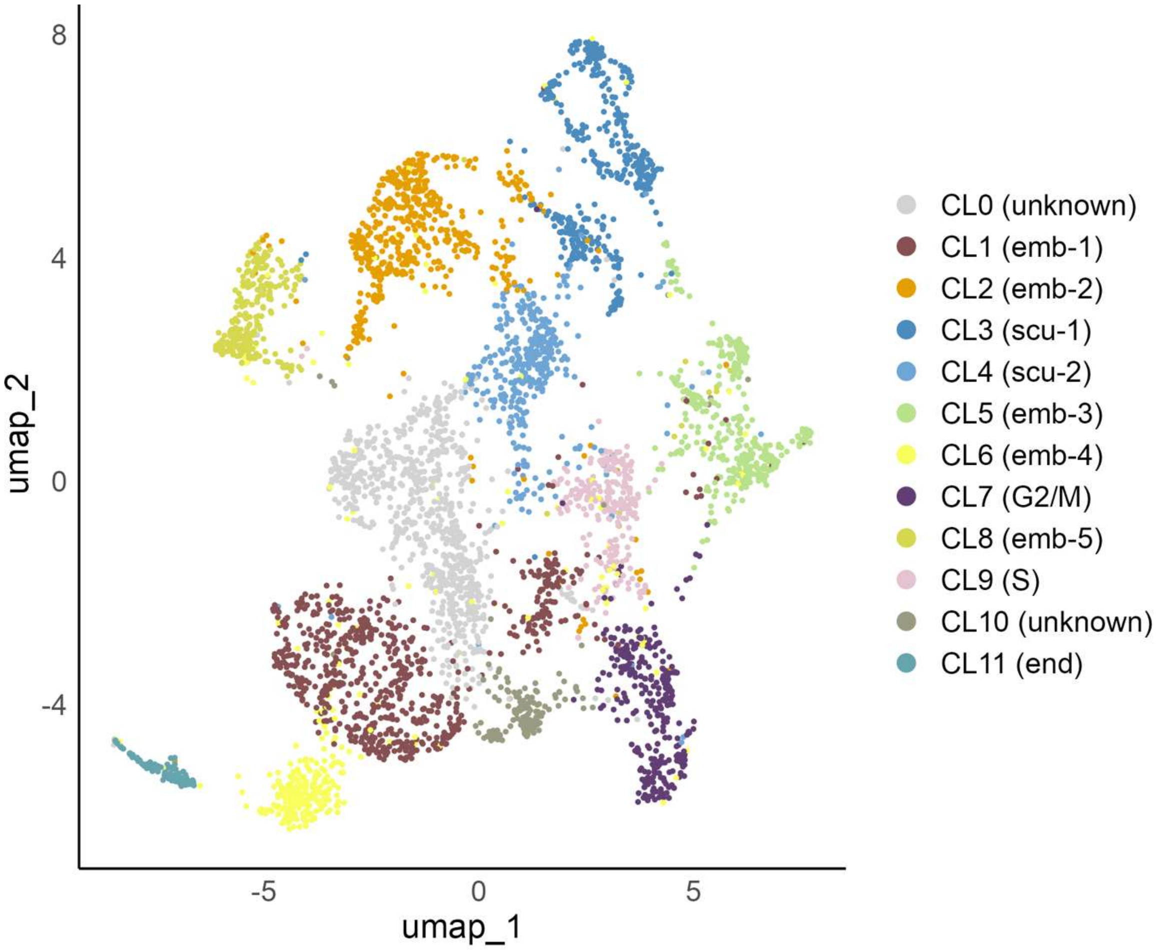

Supplementary figure S3: UMAP plot of single-nucleus data from the biological replicate 0B_1. 12 clusters were generated using a resolution of 0.5. Cells are colored according to cluster annotations.

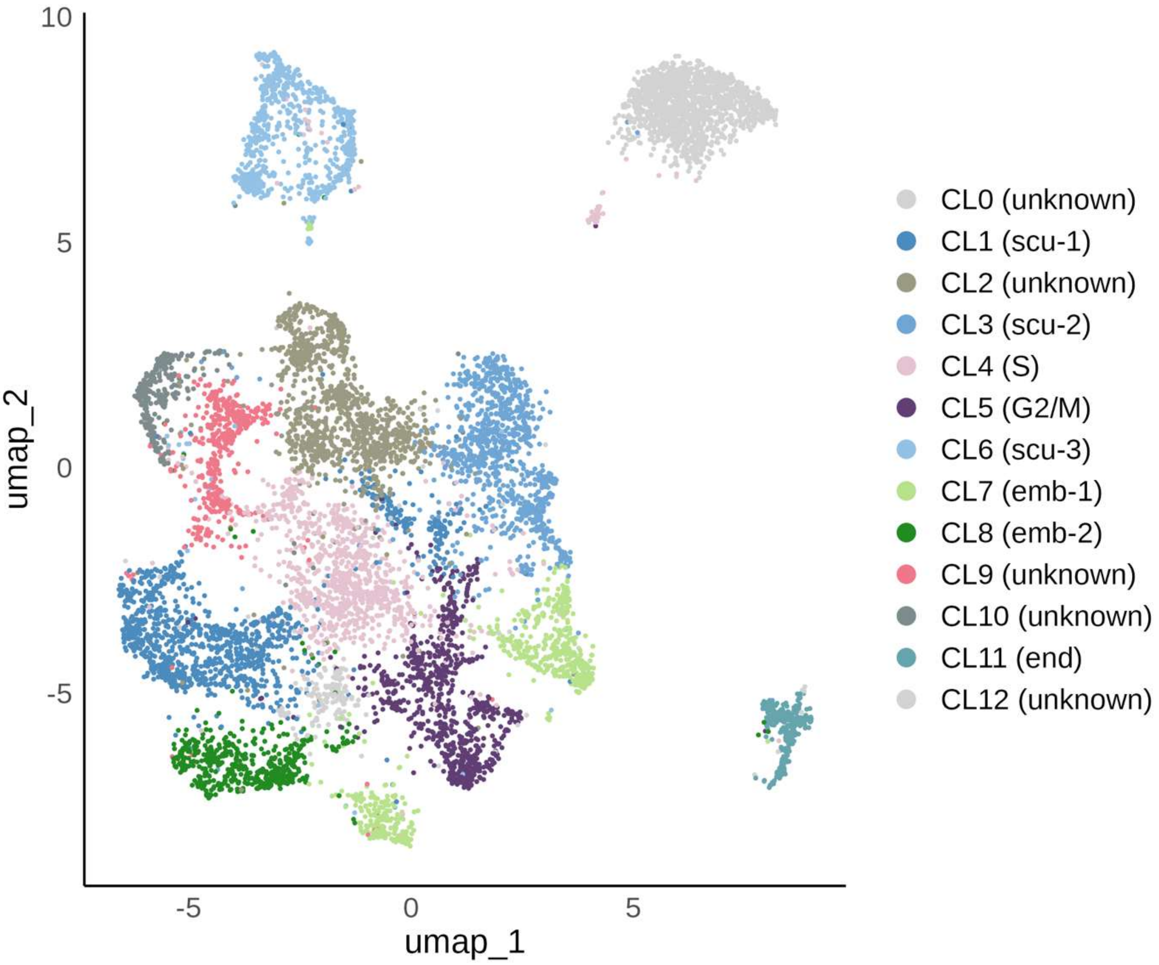

Supplementary figure S4: UMAP plot of single-nucleus data from the biological replicate 0B_2. 13 clusters were generated using a resolution of 0.5. Cells are colored according to cluster annotations.

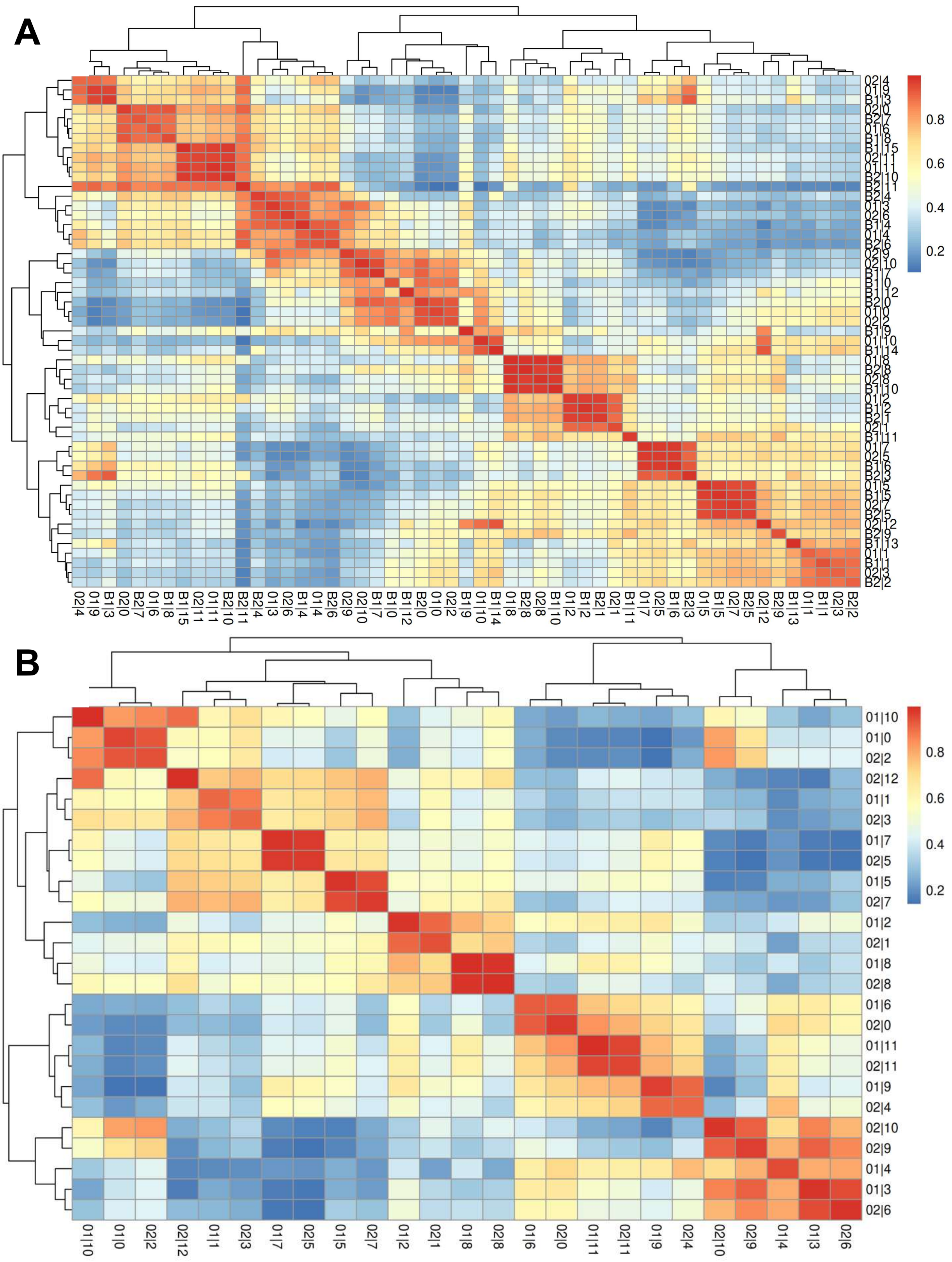

A
0.8
0.6
0.4
0.2
02|4
01|9
B1|3
02|0
B2|7
01|6
B1|8
B1|15
02|11
01|11
B2|10
B2|11
B2|4
01|3
02|6
B1|4
01|4
B2|6
02|9
02|10
B1|7
B1|0
B1|12
B2|0
01|0
02|2
B1|9
01|10
B1|14
01|8
B2|8
02|8
B1|10
01|2
B1|2
B2|1
02|1
B1|11
01|7
02|5
B1|6
B2|3
01|5
B1|5
02|7
B2|5
02|12
B2|9
B1|13
01|1
B1|1
02|3
B2|2
B
0.8
0.6
0.4
0.2
01|10
01|0
02|2
02|12
01|1
02|3
01|7
02|5
01|5
02|7
01|2
02|1
01|8
02|8
01|6
02|0
01|11
02|11
01|9
02|4
02|10
02|9
01|4
01|3
02|6

C

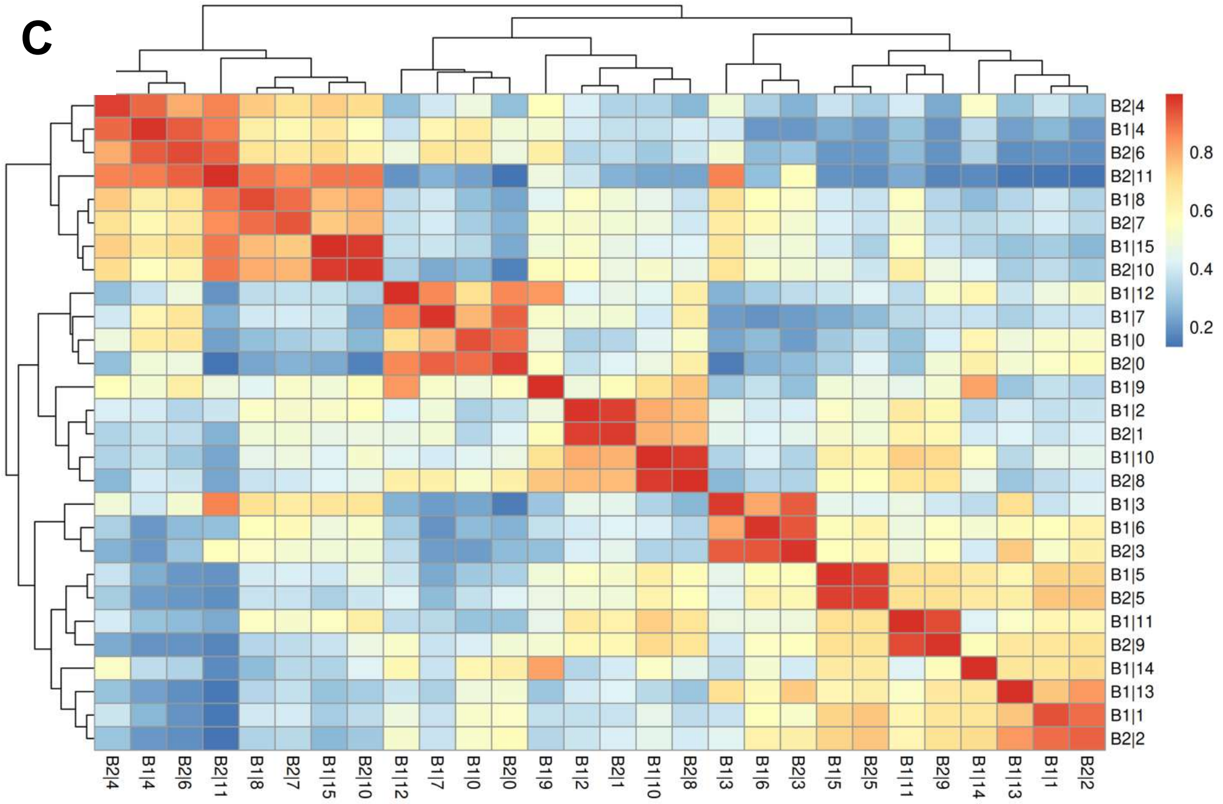

Supplementary figure S5: MetaNeighbor analysis of replicates using shared highly variable genes. Cell type similarity across four biological replicates was assessed using MetaNeighborUS with a shared set of global highly variable genes (HVGs). The resulting AUROC similarity matrix, representing the degree of cell type correspondence across replicates, was visualized as a heatmap. The heatmap color scale represents AUROC values, with red indicating higher similarity (higher AUROC scores) and blue indicating lower similarity (lower AUROC scores). **A.** Full analysis including all four replicates. **B** Two control 0B replicates. **C.** Two +B replicates. Notably, the B-associated clusters CL11 in +B_1 and CL9 in +B_2 show strong mutual similarity, where B+ samples are analyzed separately, but this relationship is not retained in the combined four-replicate analysis (A) indicating that this clustering signal is specific to the B+ context and is diluted when all replicates are analyzed together.

A

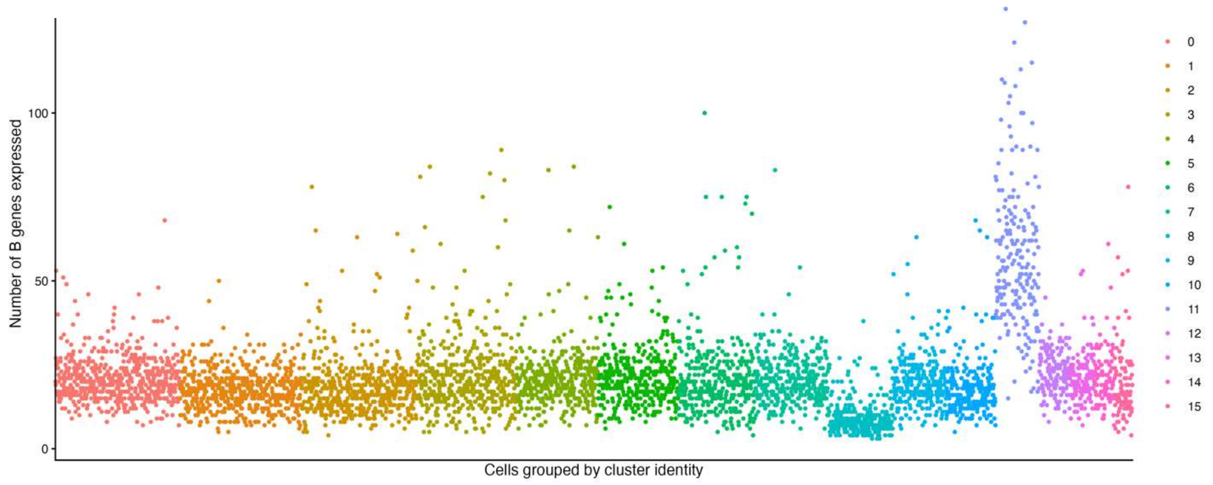

B

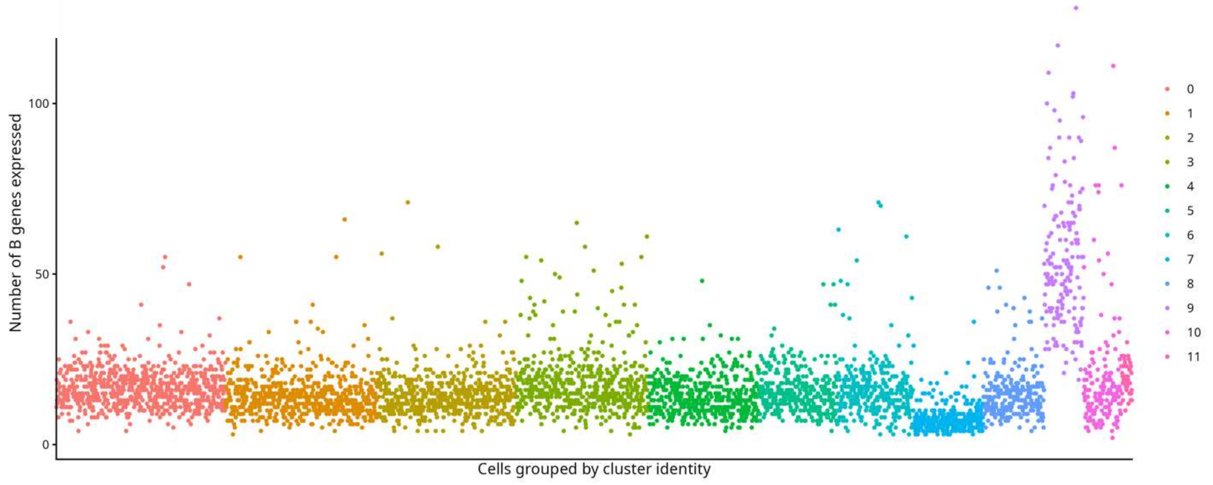

Supplementary figure S6: Distribution of B gene expression per cell across Seurat clusters in replica +B_1 (**A**) and +B_2 (**B**). Each dot represents a single cell, colored by cluster identity and ordered by cluster along the y-axis. The x-axis shows the number of high confidence B genes with detectable expression (non-zero SCT values) per cell.

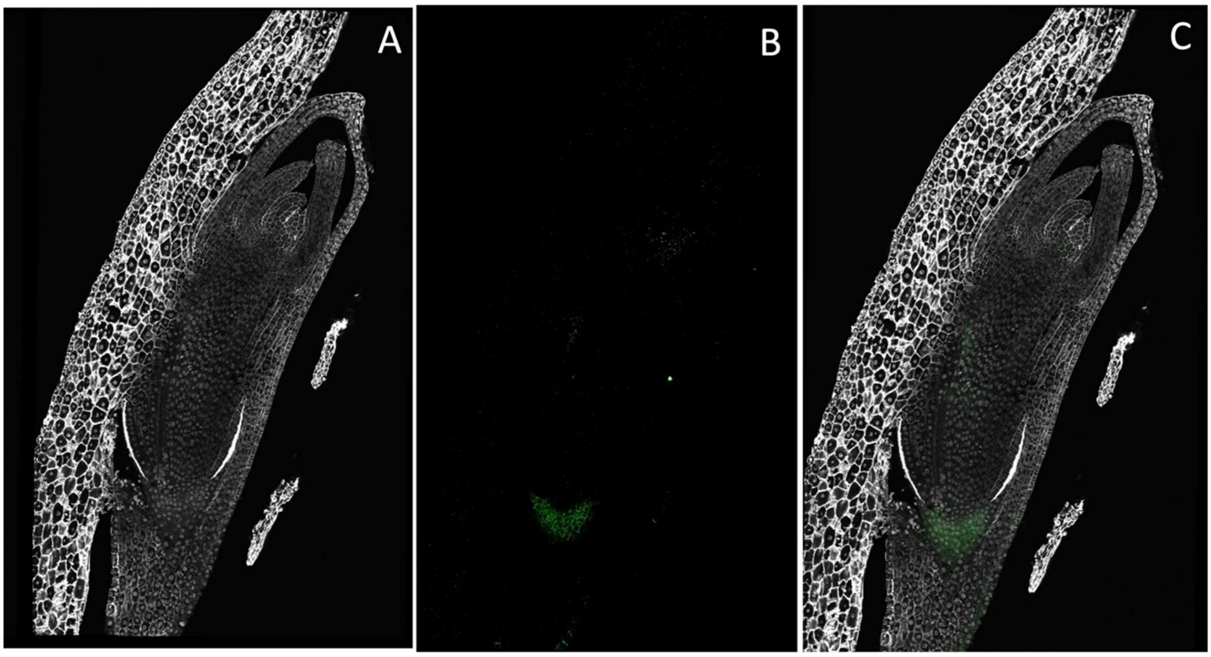

Supplementary figure S7: Whole +B embryo cryosection with gene transcript g32312 hybridized. **A** – embryo counterstained with DAPI, **B** – detection of 488-labeled RNA probe, **C** merged picture of both channels.

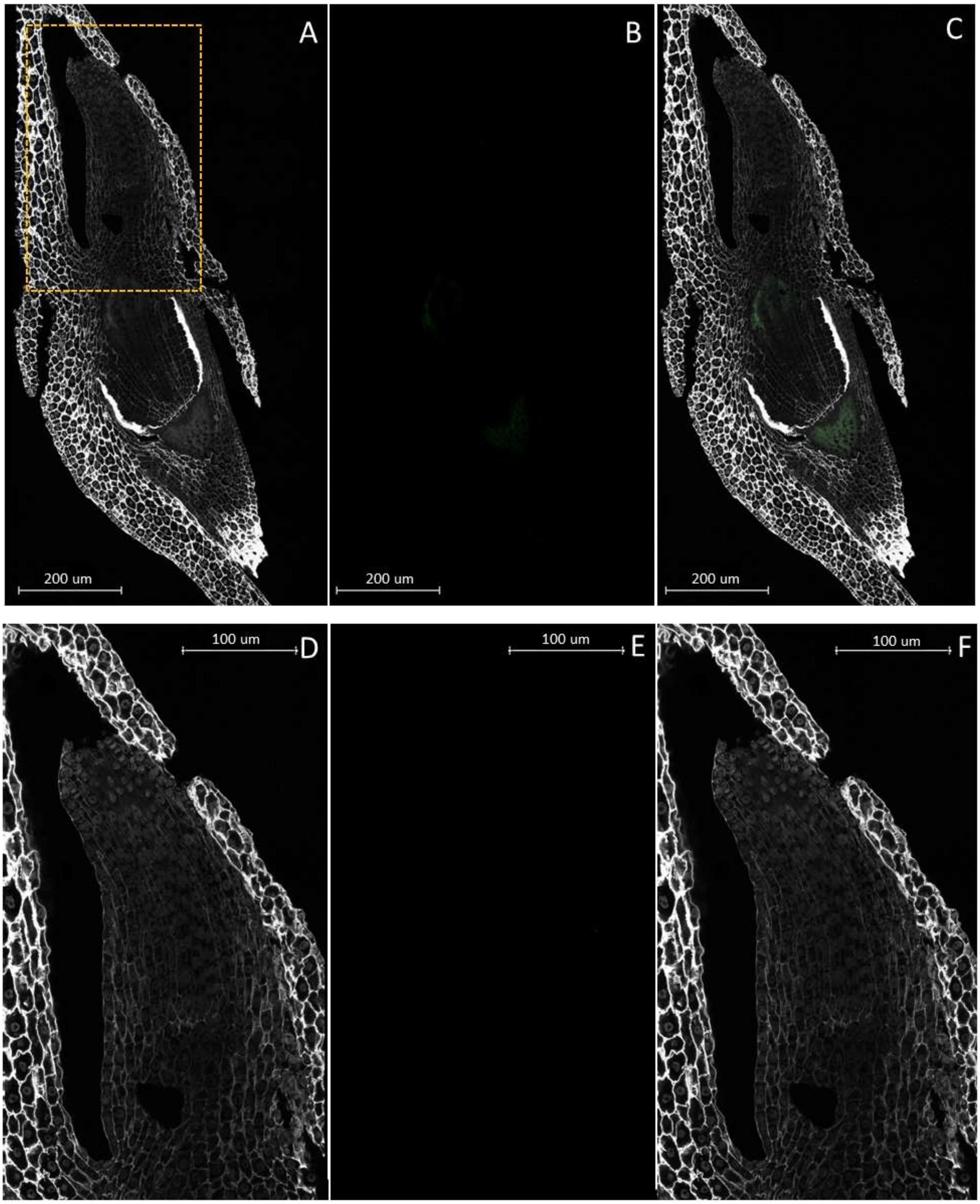

Supplementary figure S8: Localization of B-gene transcript g32312 on cryosections of embryos lacking B chromosome (negative control). Upper panel: whole embryo section. Lower panel: detail of aerial embryonic organs (area of interest in +B embryos) indicated by yellow dash line on picture A. A and D – embryo counterstained with DAPI, B and E – detection of 488-labeled RNA probe, C and F- merged picture of both channels.

A

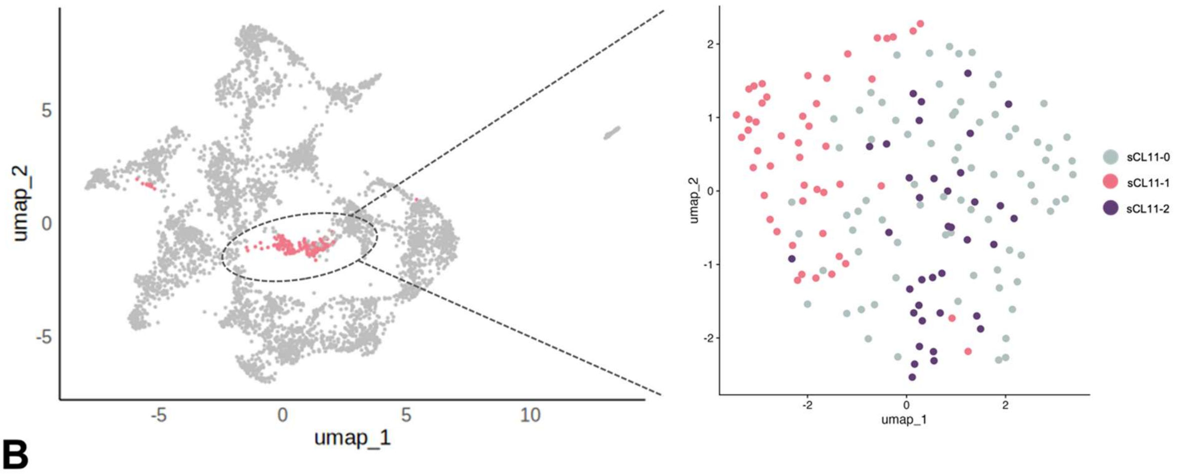

B

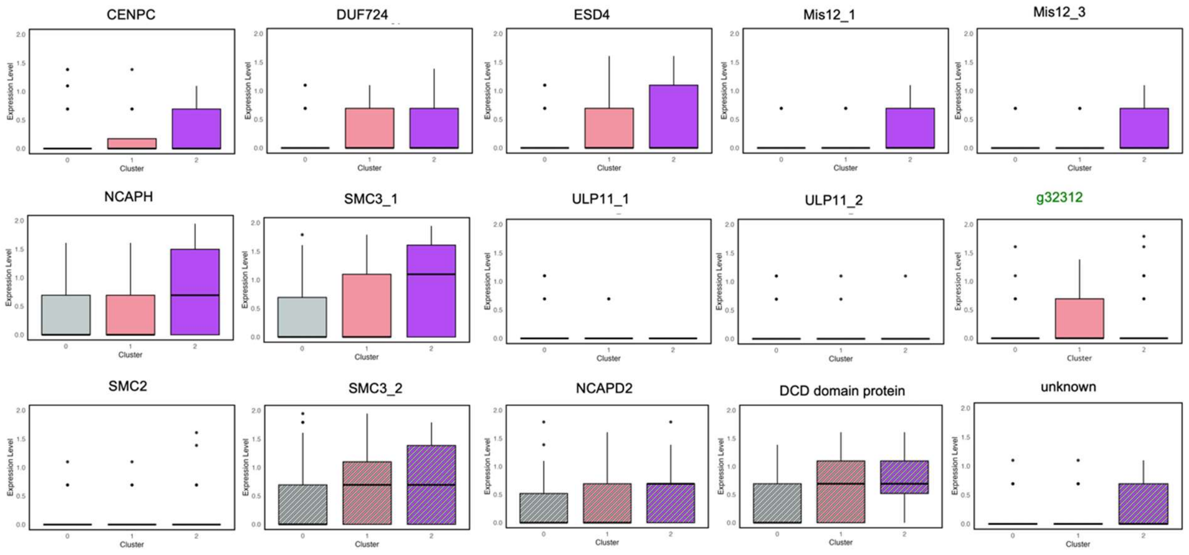

Supplementary figure S9: Analysis of a cluster associated with B chromosome elimination candidate gene expression. **A.** Left: UMAP representation of all captured single nuclei in +B_2 sample, as shown in Figure S2, highlighting the the B cluster (pink). Right: UMAP displaying independent clustering of the nuclei in the B cluster, with resolution set to 0.8. **B.** Boxplots depicting the expression levels of B chromosome elimination candidate genes in the B cluster (note elevated expression of candidate genes in subcluster sCL11-2). Boxplots for candidate genes with no detectable expression were omitted Another boxplot shows the expression of the g32312 marker gene (highlighted in green), selected for validation of the B chromosome cluster (note expression of the gene across all three subclusters). Boxplots with stripe pattern highlight newly identified B chromosome elimination candidate genes across subclusters.
